# Effect of Benzoic Acid on Nutrient Digestibility and Rectal Microbiota of Weaned Holstein Dairy Calves

**DOI:** 10.3390/ani15142080

**Published:** 2025-07-14

**Authors:** Haonan Dai, Dewei Du, Qi Huang, Jia Guo, Shujing Li, Wenli Yu, Zengyuan Zhao, Peng Sun

**Affiliations:** 1 State Key Laboratory of Animal Nutrition and Feeding, Institute of Animal Science, Chinese Academy of Agricultural Sciences, Beijing 100193, China; daihaonan0325@163.com (H.D.); dudw120459@163.com (D.D.); workhq@163.com (Q.H.); guojia_work@163.com (J.G.); 2 Shijiazhuang Tianquan Elite Dairy Ltd., Shijiazhuang 050200, China; embryochina@163.com (S.L.); anboyuwenli@126.com (W.Y.); zhaozengyuanjsdx@163.com (Z.Z.)

**Keywords:** Holstein dairy calf, benzoic acid, nutrient digestibility, rectal microbiota

## Abstract

Our previous study has shown that supplementation of 0.50% benzoic acid (BA) increased growth performance, promoted rumen fermentation, and improved the composition and function of rumen microbiota. This research was designed to conduct a deeper exploration of the impacts of dietary supplementation with BA on the apparent digestibility of nutrients and the composition of rectal microbiota in weaned Holstein dairy calves. Sixteen Holstein heifer calves with similar body weights (91.2 ± 0.7 kg) were selected and randomly allocated into two groups, each comprising eight calves. Calves in the control group (CON group) were fed with a basal diet, while those in the benzoic acid group (BA group) were fed with the basal diet supplemented with 0.50% benzoic acid (on a dry matter basis). The experimental period started at 60 days of age and ended at 102 days of age, lasting for a total of 42 days. The calves were weaned at 60 days of age, with a transition period of 7 days. Feed samples were collected every two weeks, fecal samples were collected from 99 to 101 days of age, and blood samples were collected at 102 days of age. The results showed that supplementation with BA did not influence the digestibility of dry matter, crude protein, ether extract, neutral detergent fiber, acid detergent fiber, calcium, and phosphorus between the two groups. Compared with the CON group, BA supplementation tended to decrease the total cholesterol (TC) in the serum of the calves (*p* = 0.067). Supplementation with BA increased the relative abundances of the two beneficial bacteria, *Bifidobacterium* and *Bifidobacterium pseudolongum* (*p* < 0.05, LDA > 2), but decreased that of the harmful bacterium, *Clostridium sensu stricto 1*, in the rectum of dairy calves. The microbial functional prediction revealed that the fecal microbial metabolism involved in primary bile acid biosynthesis was higher in the calves from the BA group. In conclusion, the present study demonstrated that adding 0.50% BA to the diet did not influence the apparent nutrient digestibility, but improved rectal microbiota health, which finally promoted the growth performance in weaned Holstein dairy calves.

## Simple Summary

Our previous study has shown that supplementation of 0.50% benzoic acid (BA) increased growth performance, promoted rumen fermentation, and improved rumen microbiota’s composition and functional traits. This study further evaluated supplementation of 0.50% BA on the apparent nutrient digestibility and rectal microbiota of weaned Holstein dairy calves. The results showed that BA supplementation had no impact on apparent nutrient digestibility, but it improved intestinal health by increasing the relative abundances of *Bifidobacterium* and *Bifidobacterium pseudolongum,* while decreasing that of *Clostridium sensu stricto 1*, and, finally, it enhanced primary bile acid biosynthesis. This study indicates that BA may be an effective additive in the rearing of dairy calves during and after weaning.

## 1. Introduction

Early weaning is profitable during the raising of dairy heifer calves due to the decrease in raising cost and the consumption of milk by calves, which in turn increases the amount of milk available for human consumption [1,2]. However, the changes, such as feeding methods and feed materials, may cause obvious stress responses in calves during the early weaning period, resulting in weaning stress, as the digestive organs of calves are still developing, and their resistance to the external environment is weak [3,4]. It is known that weaning stress always affects the growth performance, feed utilization, rumen development, and intestinal health of dairy calves [5,6,7]. Thus, the mitigation of weaning stress in calves represents a pressing issue that requires immediate resolution within the developmental trajectory of modern, large-scale, and intensive dairy farming operations. Except for selecting an appropriate weaning age and appropriate weaning methods, feeding appropriate feed additives in moderation is also an effective method [8,9].

Organic acids, a new type of feed additives characterized by their carboxyl (R-COOH), have been widely used in animal production for their ability to enhance growth performance and intestinal health, and are expected to be a potential substitute for antibiotics [10,11]. Benzoic acid (BA), an aromatic carboxylic acid featuring the most simplistic molecular structure, serves as a common food preservative and has also been used as a feed additive for livestock and poultry [12,13,14]. In the early years of 2005 and 2017, respectively, the EFSA Panel on Additives and Products or Substances Used in Animal Feed (FEEDAP) released two opinions addressing the efficacy and safety of BA when used as a feed additive for fattening piglets and weaned pigs [15]. In China, BA has been listed in the *feed additive variety catalog* since 2013, allowing it to be applied to all breeding animals, but its dosage is not specified [16].

As mentioned above, many previous studies have confirmed the beneficial effect of BA on monogastric animals [12,13], but limited reports have been available on ruminants, especially for dairy calves during and after weaning. Our previous study has shown that supplementation of 0.50% BA effectively promoted the growth performance, optimized rumen fermentation parameters, and modulated both the composition and functional profiles of the rumen microbiota in weaned Holstein dairy heifer calves [17]. However, further study is still required to investigate the effects of appropriate dosage of BA supplementation on apparent nutrient digestibility, blood biochemistry, and rectal microbiota of weaned Holstein dairy calves to comprehensively evaluate its bioavailability, and further validate the scientific rationale behind the 0.50% BA supplementation level, which is anticipated to offer insights for the utilization of BA in the rearing of Holstein dairy calves during the early weaning phase.

## 2. Materials and Methods

The animal protocol for the present study was approved by the Chinese Academy of Agricultural Sciences Animal Care and Use Committee (protocol number IAS2023-138). All the animals were raised and cared for in accordance with the “Guidelines for the Management and Use of Laboratory Animals” of the Institute of Animal Science, Chinese Academy of Agricultural Sciences (IAS, CAAS).

### 2.1. Animals, Diets, and Experimental Design

This research was carried out at Xingtai Junchang Pastoral Industry Co., Ltd. (Xingtai, Hebei Province, China). In this study, 16 healthy Holstein heifer calves, all 60 days of age and with a comparable body weight of 91.2 ± 0.7 kg, were chosen and randomly assigned into two groups, each comprising 8 calves, using a random number generator. Calves in the control group received a basal diet, whereas those in the BA group were fed with the basal diet supplemented with 0.50% BA (on a dry matter basis). The BA with 100% purity was obtained from Guangzhou Huayu Biotechnology Co., Ltd. (Guangzhou, China), as described in our previous study [17].

Calf feeding management has been described in our previous study [17]. The newborn calves were immediately transferred to the nursery pen after they were fed with 4 L of colostrum within 1 h after birth. For the next two days, colostrum was fed three times a day, with 2 L each time. At 3 days of age, they were transferred to the calf hutches from the nursery pens. The calf hutch was 3.7 m in length, 1.4 m in width, and 1.5 m in height, and enclosed by iron railings and bedded with regularly replaced wheat straw. From 4 days of age, the calves were administered 8 L of heated raw milk along with starter. From 21 days of age onward, moderate amounts of oat grass were offered to the calves. At the age of 60 days, the calves started weaning, until their cessation at 67 days of age. During this period, the daily milk feeding volume was gradually decreased by 1 L. At 68 days of age, the calves were continuously provided with a growth feed specifically formulated to satisfy the nutritional requirements of weaned calves, serving as a replacement for the starter feed. The growth feed was administered three times daily at 08:30, 14:30, and 21:30. The experiment spanned 42 days, commencing on day 60 and concluding on day 102. Throughout the experimental period, the calves had free access to feed and water. The basal diet’s composition and nutrient levels adhered to or exceeded the nutritional recommendations outlined in the National Research Council (NRC, 2021) [18] are shown in Table 1.

### 2.2. Sample Collection and Analysis

#### 2.2.1. Feed and Feces Sampling and Analysis

During the experiment, feed samples were collected every two weeks using the quartering method and stored at 4 °C [19]. According to the standard operating procedures required by the Association of Official Analytical Chemists (AOAC), the dry matter (DM), crude protein (CP), ether extract (EE), calcium, and phosphorus of the feed were determined using the method specified in AOAC (2005; method 930.15) [20], AOAC (2000, method 976.05) [21], AOAC (2003, method 4.5.05) [22], AOAC (1990; method 985.35) [23], and AOAC (1990; method 986.24) [23], respectively. The determination of neutral detergent fiber (NDF) and acid detergent fiber (ADF) referred to the method proposed by Van Soest et al. [24].

From days 99 to 101, fecal samples were collected from each calf every 6 h. The calves were stimulated to defecate, and about 300 g of feces was collected each time and stored at −20 °C. After collection, the feces taken from each calf were pooled, mixed, and subsampled, and were divided into 2 portions. Then, one subsample of the feces was added to 10% (*vol*/*vol*) 6 M HCl for nitrogen analysis, and the other was stored at −20 °C for the analysis of DM, EE, NDF, ADF, Ca, and P. Acid-insoluble ash was determined in feed and fecal samples, as described by Zhong et al. [25], to estimate the apparent total-tract digestibility.

Another fecal sample was taken from the rectum of each calf on the morning of day 102. Stainless-steel trays (20 × 27 × 2 cm) and centrifuge tubes (Corning, NY, USA) were sterilized using an autoclave and UV light (Longpro Co. Ltd., Guangzhou, China) before the collection of the samples, respectively. The fecal samples were sorted and marked in the trays and collected into the aseptic centrifuge tubes. These fecal samples intended for microbiota analysis were promptly snap-frozen in liquid nitrogen and subsequently stored at −80 °C.

After completing the detection of the nutritional levels of the diet and rectal feces, the apparent nutrient digestibility was calculated using the following formulas:

Apparent nutrient digestibility (%) = [1 − (insoluble ash content of hydrochloric acid in feed × the nutrient content in the feces)/(the hydrochloric acid insoluble ash content in the feces × the nutrient content in the feed] × 100.

#### 2.2.2. Blood Sampling and Determination

On the morning of day 102, after weighing each calf, blood samples were collected via jugular vein puncture into 10 mL vacutainer tubes containing heparin anticoagulant. The samples were centrifuged at 4 °C for 15 min at 3000× *g*, then the supernatant was aspirated and aliquoted into 2 mL cryotubes, and the resulting plasma was stored at −80 °C for subsequent analysis.

The contents of plasma total cholesterol (TC), triglyceride (TG), aspartate aminotransferase (AST), alanine aminotransferase (ALT), alkaline phosphatase (ALP), total protein (TP), albumin (ALB), globulin (GLB), glucose (GLU), and blood urea nitrogen (BUN) were determined using a Mindray BS-420 automatic biochemical analyzer (Mindray, Shenzhen, China). The specific methods were strictly in accordance with the instruction manual.

#### 2.2.3. DNA Extraction, PCR Amplification, and 16S rRNA Gene Sequencing

Under the accession number PRJNA1199892 (https://www.ncbi.nlm.nih.gov/sra/PRJNA1199892, (accessed on 24 December 2024)), all samples’ 16S rRNA sequencing data were submitted to the NCBI Sequence Read Archive (SRA).

The total microbial DNA was extracted from rectal fecal samples in accordance with the manufacturer’s instructions, utilizing the YH Soil DNA Kit (Majorbio, Shanghai, China). The DNA concentration and purity were quantified utilizing the NanoDrop 2000 spectrophotometer (Thermo Fisher Scientific, Waltham, MA, USA). Via 1% agarose gel electrophoresis, the integrity and quality of the extracted DNA were further assessed and confirmed.

Amplification of the bacterial 16S rRNA V3-V4 region was conducted using the GeneAmp 9700 PCR thermocycler (ABI, Los Angeles, CA, USA) with the primers 338F (5′-ACTCCTACGGGAGGCAGCAG-3′) and 806R (5′-GGACTACHVGGGTWTCTAAT-3′) [26]. The PCR reaction was carried out in triplicate. The reaction mixture and cycling conditions referred, respectively, to the methods of Wang et al. [27] and Dai et al. [13].

Following PCR amplification, amplicons were gel-extracted from a 2% agarose gel, and the AxyPrep DNA Gel Extraction Kit (Axygen Biosciences, Union City, CA, USA) was used for further purification. The QuantiFluor-ST (Promega, Madison, WI, USA) was employed to measure the DNA concentration. Following purification, the amplicons were combined at equimolar concentrations, then sequenced in a paired-end manner (PE250) on the NextSeq2000 platform (Illumina, San Diego, CA, USA) following the standard protocols.

Raw reads were processed with QIIME 1.9.1 (Denver, CO, USA), quality-filtered (scores < 20, reads < 200 bp, ambiguous bases, primer mismatches removed), and then spliced by FLASH 1.2.11 (Baltimore, MD, USA). Operational taxonomic units (OTUs) were clustered at ≥97% similarity using UPARSE 7.0.1090 (San Diego, CA, USA) [28], with taxonomy assigned via RDP Classifier 2.11 (East Lansing, MI, USA) against SILVA Database 138 (Bremen, HB, Germany). Alpha diversity (Ace, Chao, Coverage, Shannon, Simpson, Sobs) and β-diversity (PCoA via Bray–Curtis) were analyzed using Mothur 1.30.2 (Michigan, MI, USA).

#### 2.2.4. Statistical Analysis

The raw data were preliminarily sorted out by Excel 2019 (Microsoft, Redmond, WA, USA) and subjected to statistical analysis via *t*-test in SAS 9.4 (SAS Institute Inc., Cary, NC, USA). Using FastTree 2.1.3, phylogenetic trees were inferred via the Maximum Likelihood (ML) method and subsequently visualized with R software version 3.3.1. The Kruskal–Wallis H test method and stats package in R software 3.3.1 were used to analyze the differences in alpha diversity indices. Differential microbial analysis was conducted using LEfSe 1.0 (http://galaxy.biobakery.org/, (accessed on 31 May 2025)), selecting taxa with *p* < 0.05 and LDA > 2. KEGG functional prediction analysis (pathway, module, and KO levels) was conducted using PICRUSt2 2.2.0-b. Results were presented as the mean ± standard error of the mean (SEM), with *p* < 0.05 indicating a statistical significance and 0.05 ≤ *p* < 0.10 indicating a trend towards a significant difference.

## 3. Results

### 3.1. Nutrient Digestibility

As shown in Table 2, no significant differences were observed in the digestibility of DM, CP, EE, NDF, ADF, Ca, and P of the dairy calves in the BA group, compared with the CON group (*p* > 0.05).

### 3.2. Blood Biochemistry

No significant differences were observed in TG, AST, ALT, ALP, TP, ALB, GLB, GLU, and BUN in the plasma of dairy calves between the groups (CON and BA) (*p* > 0.05, Table 3). However, the TC concentration in the plasma of dairy calves in the BA group exhibited a tendency to decrease relative to that in the CON group (*p* = 0.067).

### 3.3. Rectal Microbial Compositions

Compared with the CON group, supplementation of 0.50% BA did not influence the β-diversity of the calves in the BA group (R = 0.0345, *p* > 0.05, Supplementary Figure S1). The coverage index was all above 0.99, which could well reflect species diversity and community structure. However, no significant differences were observed in the α-diversity (Chao, Shannon, Simpson, Sobs, and Ace indices) between the CON group and BA group (*p* > 0.05, Supplementary Table S1).

**Figure 1:**
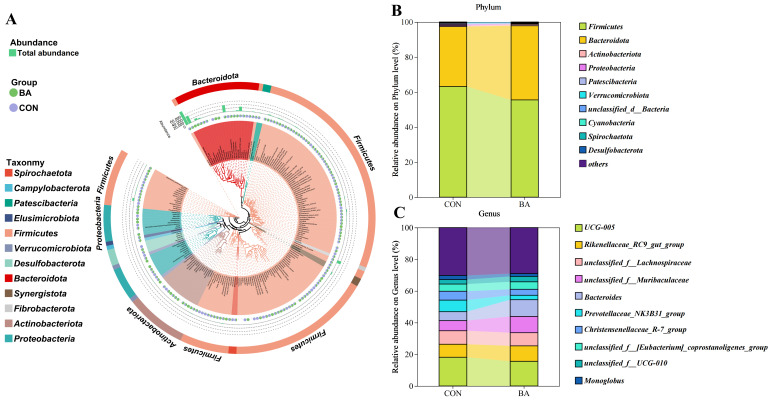
The overview of rectal microbial composition of weaned Holstein dairy calves. (**A**). Taxonomic and phylogenetic trees constructed via 16S rRNA gene sequencing of the gut microbiome. (**B**). Community biplot analysis at the phylum level reported as the percentage of relative abundance. (**C**). Community biplot analysis at the genus level reported as the percentage of relative abundance.

Twelve distinct phyla were detected at the phylum level, with the predominant bacterial phyla being *Firmicutes*, *Bacteroidetes*, and *Actinobacteriota*. The dominant bacterial genera were *UCG-005*, *Rikenellaceae Rc9 gut group*, *unclassified f Lachnospiraceae*, *Bacteroides*, and *unclassified f Muribaculaceae* (Figure 1A). While the bacterial taxa composition showed similarity at the phylum and genus levels between CON and BA calves, significant differences in the relative abundance of major phyla and genera were observed between the two groups (Figure 1B,C).

**Figure 2:**
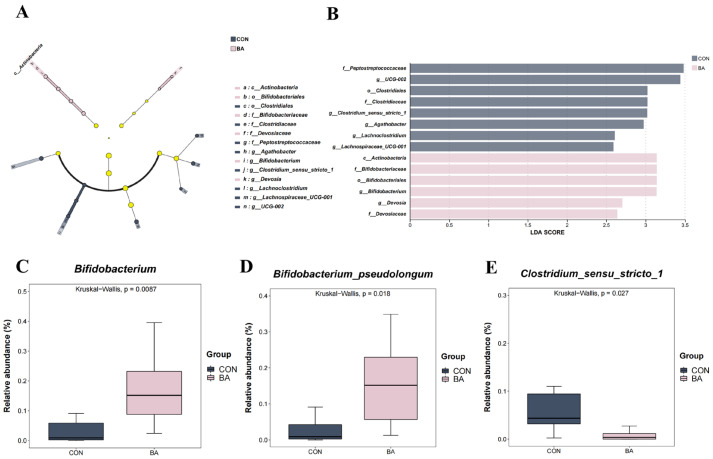
Differential analysis of microbial communities between the CON and BA groups. (**A**). Differential analysis of microbial communities between the two groups using LEfSe (*p* < 0.05, LDA > 2), accompanied by a taxonomic hierarchy plot. The yellow nodes in the figure represent no significant differences. The pink and dark blue nodes represent the microbiota with significant differences in the BA group and CON group, respectively. (**B**). LEfSe analysis of genus-level taxa with an LDA discriminant bar plot. (**C**–**E**). Genus and species with significantly different relative abundances in the BA group.

Fourteen bacterial genera that were significantly different between the two groups (*p* < 0.05, LDA > 2) were identified through LEfSe analysis (Figure 2A,B). Compared with the CON group, 0.50% BA supplementation significantly increased the relative abundances of *Actinobacteria*, *Bifidobacteriales*, *Bifidobacteriaceae*, *Bifidobacterium,* and *Devosia* in the BA group, while those of *Peptostreptococcaceae*, *UCG-002*, *Clostridium sensu stricto 1*, *Agathobacter*, *Lachnoclostridium*, *Lachnospiraceae UCG-001*, *Clostridiales*, and *Clostridiaceae* decreased significantly. At the genus and species levels, the relative abundances of *Bifidobacterium* (Figure 2C) and *Bifidobacterium pseudolongum* (Figure 2D) were significantly elevated, whereas that of *Clostridium sensu stricto 1* (Figure 2E) was significantly reduced in the BA group.

### 3.4. Functional Prediction in Rectal Microbiota

**Figure 3:**
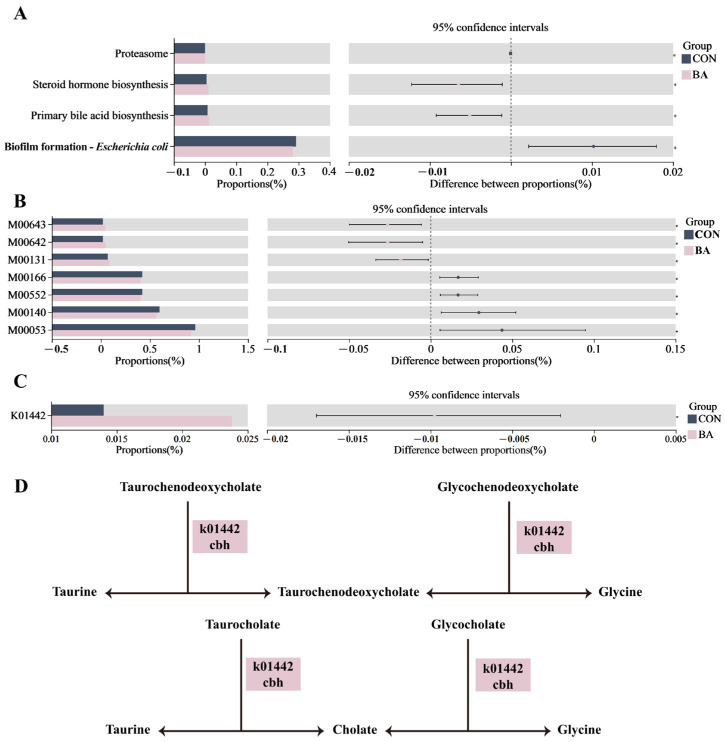
Functional prediction of the rectal microbiota in calves from the CON and BA groups. (**A**). Differential analysis of pathway between the two groups using Wilcoxon test. (**B**). Differential analysis of modules between the two groups using Wilcoxon test. (**C**). Differential analysis of KEGG Orthology (KO) between the two groups using Wilcoxon test. (**D**). Pathways involved in primary bile acid biosynthesis. The pink markers on the KO entry indicate significant increases in the BA group relative to the CON group.

Based on 16S rRNA gene sequencing data, KEGG functional prediction was performed to investigate the functional differences in the rectal microbiota between CON and BA calves. The significantly differential KEGG pathways, modules, and KO are shown in Figure 3A–C. Specifically, primary bile acid biosynthesis and the associated gene K01442 were significantly upregulated in the gut of BA-supplemented calves. Choloylglycine hydrolase (K01442) catalyzes the degradation of glycochenodeoxycholate, taurochenodeoxycholate, glycocholate, and taurocholate into taurine, chenodeoxycholate, glycine, and cholate (Figure 3D).

## 4. Discussion

Changes in feed type and feeding mode affect the calves’ physical health and gastrointestinal development during weaning, inducing weaning stress, which reduces their growth performance [1,2]. Our previous study examined how varying levels of BA supplementation influenced growth performance and rumen fermentation function, and found that 0.50% BA or more exhibited better growth and rumen fermentation-promoting effects [17]. Considering the cost saving, supplementation of 0.50% BA is optimal, which significantly increased the average daily gain (ADG) and average daily feed intake (ADFI), and finally improved the final body weight (FBW) of the dairy calves [17].

The down-regulation of genes associated with digestive enzymes, which is induced by weaning stress, leads to inadequate secretion of digestive enzymes in the gastrointestinal tract of ruminants following weaning [29,30]. This, in turn, reduces the feed utilization efficiency and affects the development of the gastrointestinal tract [29,30]. In addition, the feed for young ruminants usually has a high dietary acid-binding capacity, which also affects their feed utilization efficiency [31]. Diao et al. [32] indicated that adding BA to the diet reduces the dietary acid-binding capacity, which may help improve the feed utilization efficiency by livestock and poultry. Humphrey et al. [33] found that supplementation of 0.3% BA had no significant effect on the apparent nutrient digestibility of fattening pigs, but significantly increased the rate of nitrogen retention. While Kluge et al. [34] observed no significant difference in apparent nutrient digestibility, they found that the rate of nitrogen retention increased significantly after adding 0.50% BA to the diet of piglets. Graber et al. [35] also showed that the apparent nutrient digestibility of nutrients did not change after feeding piglets with a diet containing 0.50% BA, but the rate of nitrogen retention was significantly improved. Consistent with the previous reports, in the present study, no differences were observed in the apparent nutrient digestibility of DM, CP, EE, NDF, ADF, Ca, and P after supplementation of 0.50% BA in the diet of dairy calves.

As an important lipid molecule in livestock and poultry, cholesterol plays a crucial role in lipid metabolism. It is involved in the synthesis of cell membranes, as well as the synthesis of precursors of bioactive substances, such as bile acids and steroid hormones, and is responsible for maintaining the integrity of cell structures and the stability of systemic functions [36,37,38]. Changes in the TC level in the plasma reflect the health status of livestock and poultry. An increase in TC level not only thickens the blood vessel walls, reduces blood vessel elasticity, but also releases inflammatory factors, promotes the inflammatory response, and thus impacts the health of livestock and poultry [36,37,38]. Khukhodziinai et al. [39] suggested that supplementation of BA to the diet of broiler chickens decreased the TC concentrations in the plasma of broilers at 21 days of age. In the present study, supplementation of BA tended to decrease the TC concentrations in the plasma of dairy calves, which may indicate that BA has potential in improving the vascular function and lipid metabolism. TP is a complex mixture of various proteins, mainly including albumin and globulin [40]. Albumin, predominantly synthesized in the liver, performs several functions: maintaining colloid osmotic pressure stability, protecting blood globulins, and facilitating the transport of metabolites throughout the body [41]. Globulin, known as immunoglobulin, is also mainly synthesized in the liver and plays an important role in immunity [42]. As the main components of total protein, albumin and globulin are important indicators of liver damage. The alterations in these two indicators may predispose to the onset of inflammatory diseases [41]. In the present study, 0.50% BA supplementation had no significant effects on the plasma albumin and globulin contents, indicating that supplementation of 0.50% BA did not cause damage to the liver of dairy calves.

The gut microbiota holds a pivotal position in promoting the health of both the intestine and the host. It enhances intestinal barrier integrity, strengthening the immunomodulatory capacity and facilitating metabolism [43]. Previous research indicated that supplementation of an appropriate amount of BA to the diet can contribute to the improvement of the intestinal health of livestock and poultry. This is achieved through means such as regulating the composition of the gut microbiota, modulating enzyme activity, and ameliorating the redox state [44,45,46]. As an anaerobic probiotic colonizing the animal intestine, *Bifidobacterium* is involved in various physiological activities of the organism, including digestion, absorption, and immunity. It promotes intestinal health by stabilizing the dynamic equilibrium of the gut microbiota and suppressing the growth of pathogenic bacteria [47]. Recent research has highlighted that *Bifidobacterium*, a key member of the calf gut microbiota, is essential for optimizing growth performance. It shows a significant positive correlation with both average daily gain and average daily feed intake in calves [48]. *Bifidobacterium pseudolongum* is a probiotic strain that has been identified in numerous studies on the gut microbiome. Its relative abundance has been found to be significantly decreased in dairy cows with subclinical mastitis [49]. This strain is involved in the regulation of the gut-brain axis and exhibits immunomodulatory properties [50,51]. As a pathogenic bacterium, *Clostridium sensu stricto 1* is highly associated with inflammation. An increase in its quantity indicates a disruption of intestinal symbiosis [52,53]. The levels of proinflammatory factors were positively associated with the relative abundance of *Clostridium sensu stricto 1*, as demonstrated by Wang et al. [53]. In this study, the relative abundances of two beneficial bacteria, *Bifidobacterium* and *B.pseudolongum*, were significantly increased by the dietary supplementation of 0.50% BA. In the rectum of dairy calves, while reducing that of the harmful bacterium, *Clostridium sensu stricto 1*, which may be a contributing factor to the significant improvements in the feed intake and the average daily gain.

Bile acids were originally characterized as digestive agents that facilitate the emulsification and absorption of dietary lipids and fat-soluble vitamins in the small intestine. Beyond their role in lipid digestion, bile acids exhibit potent antimicrobial activity and contribute critically to the innate immune defense of the gut [54]. Each day, several hundred milligrams of bile acids escape the enterohepatic circulation and reach the colon, where bacterial bile salt hydrolases (BSHs) rapidly deconjugate them, liberating taurine or glycine and free bile acids [55]. Species of *Bifidobacterium* are known to possess bile acid deconjugation activity [56], with *B. pseudolongum* in particular being reported to enhance bile acid synthesis [57]. In this study, the relative abundance of *Bifidobacterium* and *B.pseudolongum* in the rectal microbiota of dairy calves was increased by dietary supplementation with BA. Functional predictions revealed significant enrichment of the primary bile acid biosynthesis pathway, along with upregulation of the BSH (K01442). These results indicate that BA supplementation fosters *Bifidobacterium* colonization, thereby enhancing calf intestinal capacity for bile acid deconjugation and overall bile acid metabolism.

## 5. Conclusions

This study demonstrated that adding 0.50% BA to the diet did not affect the nutrient digestibility, but augmented the quantities of *Bifidobacterium* and *B. pseudolongum* in the rectum, and reduced the relative abundance of *Clostridium sensu stricto 1*. Furthermore, BA supplementation promoted the functional potential for primary bile acid biosynthesis in the rectum of calves. Consequently, 0.50% BA supplementation exhibits the effect of promoting intestinal health by regulating rectal microbiota, which contributed to the increased growth performance of Holstein dairy calves after weaning.

## Figures and Tables

**Table 1:** The composition and nutrient levels of the diet for weaned Holstein dairy calves.

Items	Proportion (%)	
	^2^ Starter	^3^ Growth Feed
Diet composition (as-fed basis)		
Corn	32.00	39.73
Soybean Meal	17.00	9.73
Cottonseed Meal	2.73	3.27
DDGS	13.18	13.63
Bran	10.55	16.09
Puffed Soybean Flour	2.73	
Wheat Flour	4.54	
NaHCO_3_	0.45	0.73
^1^ Premix	7.73	7.73
Oat Grass	9.09	9.09
Total	100	100
Nutrient Levels (as dry matter basis)		
Dry Matter	89.03	88.64
Crude Protein	19.13	17.63
Ether Extract	2.88	2.48
Ash	9.47	6.40
Neutral Detergent Fiber	27.92	34.57
Acid Detergent Fiber	13.36	13.27
Ca	1.10	0.97
P	0.73	0.71

^1^ Supplied per kilogram of diet: Vitamin A 800 kIU, Vitamin D_3_ 240 kIU, Vitamin E 7 kIU, Vitamin C 200 mg, Vitamin K_3_ 15 mg, Zn 11,000 mg, Mn 7000 mg, Cu 1500 mg, I 40 mg, Se 25 mg, Co 15 mg. ^2^ Starter was provided between 1 and 60 days of age. ^3^ Growth feed was provided between 61 and 102 days of age.

**Table 2:** Effect of BA supplementation on the nutrient digestibility in weaned Holstein dairy calves (n = 8).

Items (%)	Groups		*p*-Value
	CON	BA	
DM	70.89 ± 4.14	72.74 ± 3.44	0.736
CP	81.11 ± 3.56	81.18 ± 1.64	0.988
EE	80.04 ± 3.48	80.09 ± 3.18	0.993
NDF	61.22 ± 2.58	62.51 ± 2.08	0.704
ADF	57.11 ± 2.84	57.75 ± 2.16	0.859
Ca	63.50 ± 3.54	64.43 ± 2.19	0.827
P	85.98 ± 2.27	86.69 ± 1.28	0.788

CON, the control group, which was fed with the basal diet; BA, the BA group, which was supplemented with 0.50% BA in the basal diet (on a dry matter basis). DM, dry matter; CP, crude protein; EE, ether extract; NDF, neutral detergent fiber; ADF, acid detergent fiber.

**Table 3:** Effect of BA supplementation on plasma biochemistry indicators in weaned Holstein dairy calves (n = 8).

Items	Groups		*p*-Value
	CON	BA	
TC (mmol/L)	2.39 ± 0.16	2.03 ± 0.10	0.067
TG (mmol/L)	0.26 ± 0.01	0.26 ± 0.02	0.923
AST (U/L)	82.03 ± 5.0	82.88 ± 4.9	0.906
ALT (U/L)	26.12 ± 2.71	30.36 ± 4.93	0.463
ALP (U/L)	273.76 ± 31.95	263.01 ± 24.39	0.793
TP (g/L)	68.15 ± 1.22	67.40 ± 1.13	0.662
ALB (g/L)	34.07 ± 0.84	35.00 ± 0.62	0.390
GLB (g/L)	34.08 ± 1.86	32.40 ± 0.98	0.441
GLU (mmol/L)	6.38 ± 0.10	6.36 ± 0.13	0.920
BUN (mg/dL)	12.39 ± 1.36	14.06 ± 0.79	0.307

CON, the control group, which was fed with the basal diet; BA, the BA group, which was supplemented with 0.50% BA in the basal diet (on a dry matter basis). TC, total cholesterol; TG, triglyceride; AST, aspartate aminotransferase; ALT, alanine aminotransferase; ALP, alkaline phosphatase; TP, total protein; ALB, albumin; GLB, globulin; GLU, glucose; BUN, blood urea nitrogen.

## Data Availability

Under the accession number PRJNA1199892 (https://www.ncbi.nlm.nih.gov/sra/PRJNA1199892, (accessed on 24 December 2024)), all samples’ 16S rRNA sequencing data were submitted to the NCBI Sequence Read Archive (SRA).

## Supplementary Materials

The following supporting information can be downloaded at: https://www.mdpi.com/article/10.3390/ani15142080/s1, Figure S1. Changes in beta diversity at the OTU level. The *p* value was tested with ANOSIM. Table S1. Changes in alpha diversity at the OTU level.
