# Supplementary figures and images for: Effect of Benzoic Acid on Nutrient Digestibility and Rectal Microbiota of Weaned Holstein Dairy Calves
Source: Animals (Basel). 2025 Jul 14;15(14):2080. doi: 10.3390/ani15142080 (PMC12291731; doi:10.3390/ani15142080)

R=0.0354, P=0.278000

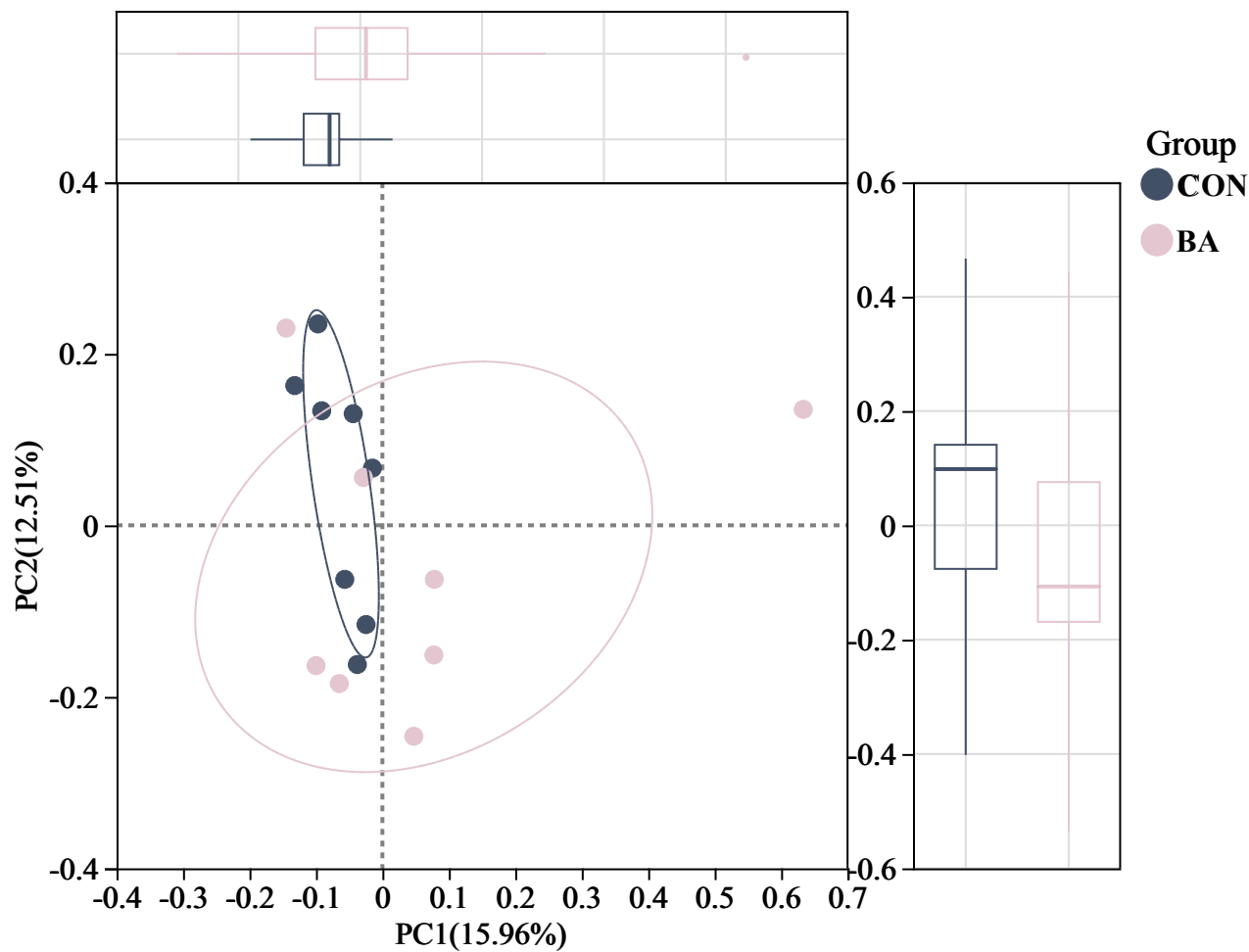

Supplement: Supplementary file 1 [file animals-15-02080-s001.zip › Supplementary figure S1.pdf]
